# Public health human resources: a comparative analysis of policy documents in two Canadian provinces

**DOI:** 10.1186/1478-4491-12-13

**Published:** 2014-02-24

**Authors:** Sandra Regan, Marjorie MacDonald, Diane E Allan, Cheryl Martin, Nancy Peroff-Johnston

**Affiliations:** 1 Western University, Arthur Labatt Family School of Nursing, Health Sciences Addition Room H34, London, ON N6A 5C1, Canada; 2 Professor and CIHR/PHAC Applied Public Health Chair, School of Nursing, University of Victoria, HSD A402, PO Box 1700 STN CSC, Victoria, BC V8W 2Y2, Canada; 3 Renewal of Public Health Systems Research Project, School of Nursing, University of Victoria, HSD B205, PO Box 1700 STN CSC, Victoria, BC V8W 2Y2, Canada; 4 Public Health Surveillance and Public Health Planning, Population and Public Health Division, BC Ministry of Health, 4-2, 1515 Blanshard Street, Victoria, BC V8W 3C8, Canada; 5 Public Health Standards, Practice and Accountability Branch, Public Health Division, Ministry of Health and Long-Term Care, 393 University Ave. Suite 2100, Toronto, ON M7A 2S1, Canada

**Keywords:** Public health human resources, Public health workforce, Policy analysis, Public health systems renewal, Public health systems research

## Abstract

**Background:**

Amidst concerns regarding the capacity of the public health system to respond rapidly and appropriately to threats such as pandemics and terrorism, along with changing population health needs, governments have focused on strengthening public health systems. A key factor in a robust public health system is its workforce. As part of a nationally funded study of public health renewal in Canada, a policy analysis was conducted to compare public health human resources-relevant documents in two Canadian provinces, British Columbia (BC) and Ontario (ON), as they each implement public health renewal activities.

**Methods:**

A content analysis of policy and planning documents from government and public health-related organizations was conducted by a research team comprised of academics and government decision-makers. Documents published between 2003 and 2011 were accessed (BC = 27; ON = 20); documents were either publicly available or internal to government and excerpted with permission. Documentary texts were deductively coded using a coding template developed by the researchers based on key health human resources concepts derived from two national policy documents.

**Results:**

Documents in both provinces highlighted the importance of public health human resources planning and policies; this was particularly evident in early post-SARS documents. Key thematic areas of public health human resources identified were: education, training, and competencies; capacity; supply; intersectoral collaboration; leadership; public health planning context; and priority populations. Policy documents in both provinces discussed the importance of an educated, competent public health workforce with the appropriate skills and competencies for the effective and efficient delivery of public health services.

**Conclusion:**

This policy analysis identified progressive work on public health human resources policy and planning with early documents providing an inventory of issues to be addressed and later documents providing evidence of beginning policy development and implementation. While many similarities exist between the provinces, the context distinctive to each province has influenced and shaped how they have focused their public health human resources policies.

## Background

The increase in communicable and non-communicable disease incidence and prevalence, changing population demographics, along with concerns about pandemics, natural disasters, and terrorism, have highlighted the challenges faced by public health systems to respond rapidly and appropriately to threats [1,2]. Governments, recognizing the importance of public health to address these threats, have focused on strengthening public health systems in many countries including Canada [3], the United States [2] and across Europe [4]. A key factor in rebuilding and sustaining public health is a robust public health workforce. Improved public health human resources (PHHR) policy, planning, and management have been identified as priorities in international reports [3,5]. In separate national public health priority setting consultations, both Canada and the United States have identified issues related to the public health workforce as priorities [6,7]. Indeed ‘(no) attempt to improve public health will succeed that does not recognize the fundamental importance of providing and maintaining in every local health agency across Canada an adequate staff of highly skilled and motivated public health professionals’ [3] (p. 136).

PHHR policy development and planning is complex and has a number of unique challenges. The myriad of sectors and stakeholders responsible for public health service delivery involve multiple levels of government – national, provincial/state, and local – along with sectors external to public health, such as primary care, schools, municipal governments, not-for-profit agencies and non-government organizations. The involvement of such a broad array of stakeholders poses challenges to coordination and planning for the right composition and distribution of PHHR [8,9]. The public health workforce includes more than 25 regulated and non-regulated health care providers [10]. Because these providers are drawn from different disciplines (both health and non-health related), there is great variation in their entry-level education. Indeed, most are prepared in generalist discipline-specific programmes with little formal public health education [9] adding more complexity to PHHR planning [11].

In Canada, national consultations have identified a vision for PHHR that includes collaborative planning, building on core public health competencies that will result in a competent and flexible workforce prepared to “meet the population’s public health needs, and reduce health and social disparities” [10] (p.iii). While national visions provide a collective direction for policy development and implementation, public health programmes are primarily developed and delivered at the provincial and local levels (for example, municipal, regional). To date, there has been little published about PHHR policy and planning at the provincial or local level and specifically during a time of change or renewal for public health systems. The purpose of this paper is to present results of an analysis of PHHR policy and planning documents for two provinces in Canada as they each implement public health renewal activities.

### Public health context in Canada

In Canada, public health is a shared responsibility between several levels of government. At the national level, the Public Health Agency of Canada (PHAC) provides national leadership for action on public health with roles in facilitating intergovernmental collaboration on public health and supporting national approaches to policy and planning [12]. While the PHAC collaborates with provincial, territorial, and municipal levels of government as well as non-government organizations, each province or territory has its own public health legislation [13,14] and unique structure for public health service delivery.

In 2003, in the aftermath of the SARS event, two Canadian provinces, British Columbia (BC) and Ontario (ON), undertook processes to define, develop, and implement public health requirements for services and programmes. The result of these parallel but independent processes led to the BC Core Public Health Functions [15] and the Ontario Public Health Standards [16]. Each province has similar and unique public health contexts. For example, in BC, public health is funded by the provincial government and delivered by five regional health authorities and one provincial health authority while in Ontario funding is shared between the provincial government and municipal (local) governments and delivered by 36 local boards of health. In addition, in both provinces some provincial agencies participate in delivery of some services. See Table 1 for additional information on BC and ON public health context.

**Table 1:** Public health context – British Columbia and Ontario

**Context**	**British Columbia (BC)**^**1**^	**Ontario (ON)**^**2**^
Public health structure	Public health units are integrated within five geographical and one provincial health authority. Public health is integrated into the larger health care system.	Public health is delivered by thirty-six individual public health unitseach with a board of health responsible for local programmes and service delivery within the larger health care system.
Governance	Provincial government and regional health authorities.	Provincial government and municipal governments.
Funding	Provincial funding to health authorities.	Provincial and municipal funding.
Provincial core policy	Core public health functions framework, with 20 core programmes implementation of which is guided by evidence reviews and model core programme papers.	Ontario public health standards with one foundational standard and 14 standards implementation of which is guided by protocols and guidelines.
Provincial public health agencies		
	Provincial health services authority, within which the British Columbia center for disease control is situated.	Ontario agency for health protection and promotion (later renamed Public Health Ontario).

Sources: ^1^http://[http://www.health.gov.bc.ca/pho/what-is-public-health.html];

^2^http://[http://www.health.gov.on.ca/english/public/program/pubhealth/public_mn.html]

### Renewal of public health systems

In this paper we report on results of a comparative policy analysis conducted within a larger programme of research. The aim of this policy analysis was to compare PHHR-relevant policies and planning documents in two Canadian provinces, BC and ON, as they each implement public health renewal activities.

The ‘Renewal of Public Health Systems (RePHS) in BC and Ontario’ is a five year nationally funded programme of research (2009 to 2014) [17]. The RePHS study seeks to answer two major research objectives:

1. To explore and understand the core public health functions implementation process and the contextual factors influencing it in BC and ON.

2. To examine and understand the impact and outcomes of core public health functions implementation at organizational, systems, and population levels in both BC and ON.

Three cross-cutting themes are also investigated in this study:

1. Public health human resources policy, planning, and management;

2. Relationships between public health and primary care sectors; and

3. Integration of an equity lens.

Together these objectives and cross-cutting themes guide an integrated programme of research. One aim of the RePHS study was to compare how each province articulated their provincial policy directions related to PHHR at the outset of this programme of research. The policy analysis reported in this paper will provide a baseline against which to compare PHHR developments at the conclusion of the RePHS programme of research.

Questions guiding this policy analysis were: What specific aspects of PHHR policy, planning, and management are discussed in BC and ON government and public health organization documents? What are the contexts in which PHHR policy, planning, and management are discussed? and To what extent are these similar/different?

## Methods

A comparative policy analysis of documents in BC and ON was conducted to understand key aspects of PHHR policy and planning during implementation of public health renewal activities. We defined policy as those ‘courses of action (and inaction) that affect the set of institutions, organizations, services and funding arrangements of the health and health care system. It includes policy made in the public sector (by government) as well as in the private sector’ [18] (p. 6). Policy analysis is the systematic and disciplined examination of policy with the objective of understanding the process, content, or outcomes of policy [18,19]. In this analysis we were interested in analysing the *content* of PHHR-relevant policy and planning documents within the context of change in public health. The research team consisted of academic researchers and government decision-makers with public health expertise in both provinces; team members are also investigators on the larger RePHS study.

### Document identification and selection

For the purposes of this study, we included provincial government and select public health organizations’ policy and planning documents relevant to public health and/or PHHR. Government and public health organization (for example, public health associations) websites in both provinces were accessed for documents and key informants and public health experts on our team identified additional relevant documents within their respective provinces. The types of documents included: provincial annual reports, core public health function/standards documents, commission reports on SARS, health human resources (HHR) and PHHR specific reports, health profession legislation, and other public health (PH) reports such as competencies development and leadership frameworks. Documents published between 2003 (beginning at the time of the SARS event) and March, 2011 were included in the analysis. Publicly available and internal provincial government and public health-related policy documents were obtained for BC (n = 27) and ON (n = 20). A small number of government documents were not publicly available but were identified as relevant to the analysis by our government team members. Relevant text from these documents was excerpted to include in the analysis while ensuring the confidentiality of documents was not compromised. While we made every effort to identify and access all relevant documents, some older documents many not have been available to the authors. See Additional file 1 for a list of all documents analysed in this study.

### Data analysis

A coding template was developed inductively, informed by two national HHR documents: *A Pan-Canadian Framework for Public Health Human Resources Planning*[10] and *A Framework for Pan-Canadian Collaborative Health Human Resources Planning*[20]. Both these documents provide conceptual frameworks describing HHR and PHHR planning. Broad categories relevant to PHHR and based on the national HHR documents were identified: background/context, responsibility/accountability for HHR planning, collaborations/partnerships, policy assumptions, HHR planning activities/elements, and HHR management. Within the broad categories, several sub-categories were developed to capture more specific detail. For example, for the category HHR planning activities/elements, sub-categories included: supply/HHR characteristics, deployment/utilization, leadership, education/training/competencies, capacity, and matching HHR to health/service requirements. For each sub category, a definition was developed to assist with consistent coding (see Additional file 2 for the coding template). Consensus on the coding categories, sub-categories and definitions was achieved with team members. A deductive content analysis was conducted using accepted methods [21] to compare key aspects of PHHR policy, planning and management including policy assumptions, collaborations, scope of practice, competencies/education, and planning approaches. NVivo 9, a qualitative software program [22], was used to organize and analyse the data. Members of the research team independently coded text within the policy documents using the coding template. After all the documents were coded and reviewed by two researchers, reports were obtained for each code, reports were reviewed by the entire the team, and consensus achieved on the coded text. A comparative analysis of BC and ON coded text was then conducted to identify similarities and differences. Narrative descriptions were written for the most frequently coded text and reviewed by the team.

## Results and discussion

We present the results and discuss the findings according to the most frequently coded HHR sub-categories across the policy documents, identifying and describing differences and similarities between provinces. Table 2 summarizes the top coded categories along with the percentage of documents coded to that category. For example, 90% of the ON documents and 59% of BC documents contained text relevant to the code *HHR Planning Activity - education/training/competencies/scope of practice*.

**Table 2:** Top codes by percentage of documents for each province

**Category/Subcategory**	**BC**	**ON**
HHR Planning activity/element: Education/training/competencies/scope of practice	59%	90%
HHR Planning activity/element: Capacity	56%	55%
HHR Planning activity/element: Supply and characteristics	29%	55%
Collaborations/partnerships: Intersectoral collaboration	30%	45%
HHR Planning activity/element: Leadership	41%	30%
Background/context: PH specific planning context	26%	45%
Background/context: Priority populations	26%	20%

Note. BC documents n = 27; ON documents n = 20.

### Education, training, competencies and scope of practice

Policy documents in both provinces discussed the importance of having an educated, competent public health workforce with the appropriate competencies (knowledge, skills and attitude) for the effective and efficient delivery of public health services. To achieve this, provinces identified the importance of understanding essential public health functions in order to align the competencies necessary to carry out public health functions with appropriate health human resources. These functions were determined through extensive consultation processes in both provinces [23,24] and nationally [25]. There are distinct differences in how BC defines essential public health functions compared to ON and Canada; but for all three, essential functions of public health include monitoring and assessment of population health status, public health surveillance, health promotion, disease and injury prevention, and health protection and enforcement [15,25,26]. The BC Core Public Health Functions Framework [15], includes two different categories of essential functions:

1. those that are unique and are specifically geared towards public health (the essence of what public health does); and

2. those that help the health system to carry out its core programmes or services. This set of functions is common to the entire health system.

Both of these are considered essential for the provision of public health programmes and services.

Within the context of the BC Core Public Health Functions framework, the essential public health functions (entitled strategies), the 20 core programmes, and the application of equity and population lenses are specific public health functions which are supported by system capacity [15]. The Ontario Public Health Standards set out the minimum requirements for public health programmes and services delivered by the 36 boards of health [16]. The Ontario Public Health Standards consist of four principles (need, impact, capacity, and partnership/collaboration), one foundational standard with specific areas (population health assessment, surveillance, research and knowledge exchange, and programme evaluation), and 14 programme standards grouped into five programme areas. The standards also set out requirements, including public health functions (assessment and surveillance, health promotion and policy development, disease prevention, and health protection) that all boards of health must implement [16].

Provincial government and public health organizations’ policy documents dated in the early period of this analysis identify the beginning of efforts to develop public health competencies [15,27] and in later documents, both provinces build on the national work identifying core competency areas: public health sciences; assessment and analysis; policy and programme planning, implementation and evaluation; partnerships, collaboration and advocacy; diversity and inclusiveness; communication; and leadership [28,29]. Both provinces undertook intensive processes to identify and explicate core public health competencies that are foundational to support public health renewal including development of core functions and public health standards.

Public health associations in both provinces played an important role in identifying core public health competencies. In ON, the Ontario Public Health Association initially established a Core Competencies Task Group in 2003 with the intent of developing a set of core competencies for public health. To avoid duplication of efforts, the Ontario Public Health Association eventually partnered in the national process to develop core public health competencies [30]. In BC, a multi-phase project was undertaken by the BC Ministry of Health and the Public Health Association of BC, funded by the Public Health Agency of Canada. The purpose was to identify and address the core and technical competencies most critical to implementing the Core Public Health Functions Framework [15] and to identify competency gaps [28]. Specific objectives of the project were to:

• Implement processes to meet the competency profile gaps identified in the needs assessment;

• Recommend appropriate education response (s);

• Identify the education/training opportunities that facilitate the development of competencies throughout the BC public health sector; and

• Develop tools for education and workforce planning [28] (p.2).

The importance of strengthening partnerships between academe and public health as an important means of ensuring that the public health workforce is adequately trained was identified in documents in both provinces [27,31]. Partnerships with universities were also noted as being vital to create programmes that are more aligned with PHHR needs and the inclusion of the education sector in PHHR planning overall. Practiced-based public health experience was highlighted as a key component of education/training programmes to prepare the public health workforce. Both BC and ON documents discussed several strategies to build education capacity in PH including the need to train new PH professionals, integrating core public health competencies in discipline-specific undergraduate education programmes, increasing the profile of PH careers and enrolment in PH programmes, increasing funding for PH education (subsidies, scholarships, and so on), expanding educational modalities (online, summer institutes), and increasing paid student internships and training placements in public health contexts [23,27,32]. In the last decade, both provinces have seen an increase in development of public health education programmes including Master’s of public health (MPH). In addition, voluntary guidelines for Canadian universities offering MPH programmes were developed in 2006 and revised in 2009 [33]. Investments have been made by the ON government to enhance training and education opportunities for physicians to specialize in community medicine and public health [34] .

Building and sustaining PHHR capacity is also dependent on continuing educational opportunities and the recruitment of new professionals into the system. Public health needs to ensure a competent and diverse public health workforce by providing ongoing staff development and skill building related to core public health competencies, including quality improvement and life-long learning programmes for staff members. Both provinces identified a number of training and education needs for the workforce that include developing knowledge and skills related to: leadership capacity, applying a population and equity lens, diversity and cultural competency, the social determinants of health, interprofessional collaboration, and use of evidence in practice. In BC, specific issues related to Aboriginal health and HHR planning were highlighted [35,36] while in ON, infection control training was identified [37,38]. These specific areas reflect contextual factors specific to each province. Consistent with arguments made by Fraser and Greenhalgh [39], the complexity of public health requires that the workforce must be capable of adapting and responding to the changing context in which they work. This capability is supported by not only identifying the competencies required to provide public health services but by moving beyond these to facilitate different ways for those in the workforce to continue to learn and build on existing competence.

### Capacity

Capacity speaks to the ability of the public health system to respond to the needs of the population. Both provinces recognized the need to increase public health capacity, both in terms of system supports/changes and resources, capability development, and leadership. They also identified the need for appropriate and sufficient staff complements at the local level to meet population health needs.

The importance of system capacity to direct public health strategies and support the implementation of programmes and services at local, regional, and provincial levels was identified in the BC Core Public Health Functions [40] and the Ontario Public Health Standards [16]. Specific to BC, system capacity included health information systems, health human resources, staff training and development, research and evaluation, quality management, programme planning and management capabilities, and core competencies [15]. A number of supports for capacity building were identified including those related to PHHR:

• Supportive attitudes, values and philosophy that foster a safe, flexible learning environment

• Supportive attitudes and structures that promote cultural sensitivity and respect for diversity across all aspects of the organization

• Supportive policies, processes and structures to optimize the application of the core competencies to achieve desired health outcomes.

• Supporting advocacy to influence systemic change.

• Provision of resources that support the appropriate application and mix of competencies within the organization

• Opportunities for training, continuing education and mentoring to acquire and enhance competencies/support the effective application of competencies

• Facilitating sustainable collaborative relationships with multiple sectors and partners.

• [40] (p.8).

While in ON, boards of health are guided by four principles in the standards that explicitly speak to public health capacity and meeting local communities’ needs. The four foundational principles are: 1) Need (addressing population health needs effectively and efficiently), 2) Impact (reducing health inequities), 3) Capacity (understanding local capacity and resources, including human resources, required to achieve outcomes), and, 4) Partnership and Collaboration (fostering partnerships to promote community capacities, that is the system includes partners such as other agencies and non-governmental organizations). As with BC, the ON Public Health Standards identify a number of supports for public health capacity building including ‘organizational structures and processes; workforce planning, development, and maintenance; information and knowledge systems; and financial resources’ [16] (p.13).

In addition to recognizing the need for sufficient and appropriate PH staffing, many Ontario documents focused on *surge capacity* and emergency response to anticipated and unanticipated events [38,41]. In their analysis of the SARS event, the Ontario SARS Commission identified the lack of surge capacity as a significant issue in the ability to respond to SARS effectively [41] and the potential to respond to future pandemic events. The different focuses between BC and ON may be explained by the ability of each province to respond to and contain the spread of SARS. Because BC was able to contain the spread of SARS before it reached epidemic proportions, it is likely that the ON public health sector viewed surge capacity as more relevant and a priority than BC did.

Documents in both provinces identified that PHHR requirements to ensure sufficient capacity included an appropriate mix of core competencies, administrative support, and mechanisms such as leadership, partnership with affiliate organizations, and training and recruitment [32,41,42]. Some systemic solutions identified in these documents included:

• Sustainable employment strategies to increase the supply of HHR

• Develop increased education and training opportunities for public health professionals

• Consolidation of smaller health units (specific to the ON context only)

• Identify and implement models for effective utilization of health human resources duringan emergency

• Systems for identification, coordination, and deployment of emergency HR resources

• System of cross-training and re-assignment and

• System of expert field support to ensure all PH practitioners have access to the expertisethey need when they need it, including more effective alignment of expertise, training, and support [35,36].

### Supply and characteristics of public health human resources

Closely aligned with *capacity* was the discussion of the supply and characteristics of PHHR. Historically HHR planning has focused primarily on staffing numbers; however, many of the PHHR policy documents have approached the issue of ‘supply’ of PHHR with a broader understanding of planning. This encompasses not only the numbers of staff, but their characteristics such as age of the workforce, and education and competencies, and incorporating issues such as the requirements to meet population health needs into planning. Policy documents published after the SARS event in ON discuss issues of ‘shortages’ of public health professionals [23,27]. Given the effects of the SARS event, other public health crises in recent years, and emerging shortages of health professionals in general, it is not surprising that early policy documents were focused on these concerns. For example, early documents discussed the shortage of key public health personnel, in particular, the need for Medical Officers of Health and those health professionals with expertise in infectious diseases [23,27,41]. In contrast with ON, BC documents were less focused on the discussion of shortages and improving PHHR supply with the exception of one specific recommendation of enhancing Aboriginal PH staff [35]. At the time of the policy analysis, BC had undertaken planning for a project to enumerate the public health workforce in order to ‘to characterize, in a consistent manner, the size, composition and distribution of the formal public health system workforce among multiple health authorities and at the provincial level’ [43] (p.1).

In ON, PHHR supply was oriented more generally at systemic issues of resource availability and public health infrastructure. There was an explicit recognition that a dedicated, diverse workforce was required for optimal public health functioning; however, the documents also identified concerns about work environments and their impact on staff [27]. This is consistent with a number of studies and reports that identify the importance of quality work environments on staff recruitment and retention [44,45].

Several documents in both BC and ON offered solutions to PHHR shortages, including improving PHHR planning, increased attention to recruitment and retention strategies, increased attention to enrolment and training opportunities, and targeting strategies to address shortages of specific roles in public health [27,46-48]. In general, policy documents were more focused on the need for appropriately educated PHHR rather than simply the numbers.

### Collaborations/partnerships - intersectoral

Documents from both provinces recognize that collaboration is necessary across levels of government, within governments, between public health and primary care sectors, as well as with those beyond the health sector such as schools, communities, and other organizations. Integration of public health programmes and policies were deemed necessary to address the interrelatedness of social, physical, and mental health concerns [16,28].

BC documents explicitly discussed the importance of intersectoral, multi-sectoral and multi-disciplinary collaboration and integration in core programme papers as a strategy to ensure consistent, seamless services and achieve core functions [49-51]. In addition, the importance of collaboration between public health and primary health care was emphasized as it relates to the delivery of chronic disease prevention programmes and services [49]. BC’s focus on intersectoral and multi-sectoral collaboration, as well as collaboration between public health and primary care may relate to the fact that public health is integrated into the larger health care system whereas in ON, public health is separate from the health care system at large. Public health units are not part of the Local Health Integration Networks (regional health authorities) in ON. Public health in the BC core public health documents is viewed as being provided not just by traditional public health workers, but by other professionals working across all areas of the health care system as well as outside the health care system.

ON documents discussed the need to develop and optimize strategic relationships and collaboration with current initiatives, structures, and professional organizations, and actively engage with partners in government, local public health, health care, non-profit, industry, and academia [38,41]. Clearly defining roles and responsibilities, particularly as they relate to government and public health units, was identified as a strategy to address issues that arise in the case of infectious disease outbreaks, where the potential for conflict and procedural/ administrative holdups might be avoided with more explicit assignment of roles between sectors/jurisdictions [27]. While earlier documents discussed these policy issues and made recommendations for action, more recent documents suggest some policies related to intersectoral collaboration have been developed and implemented. For example, to address the greater coordination and consistency between sectors necessary for infection control, Regional Infection Control Networks have been established in ON [37].

### Leadership

A noted issue in public health post-SARS was the leadership deficit. Both provinces have identified the development of leadership competencies, enhanced leadership skills training, and building leadership capacity as strategic policy directions [24,27]. Leadership competencies were understood to be essential to the application of other core public health competencies and to public health worker retention, morale, and productivity [52]. For both provinces, documents outlined the roles and responsibilities of health authorities, the organizational structures of public health bodies, and relationships among public health leadership such as Medical Health Officers [13,27,31].

ON documentation more explicitly addressed a perceived lack of resources for leadership in public health, and identified the need for the province to develop resources such that health unit workers are supported in developing leadership skills [27,38]. The ON documents also spoke to the barriers, such as time, opportunities, and recognition, to developing strong leaders and leadership skills [23,29,38]. Documents indicated the need for leadership training (and valuation thereof) and professional networks. One document pointed to the importance of employing and retaining professional practice leaders (for example, nurses) in each health unit, and of identifying and cultivating good leaders [27]. Since this time, ON has implemented a new provincial initiative requiring boards of health designate a Chief Nursing Officer in each public health unit [53].

### Public health specific planning context

In terms of PH planning context, both provinces identified the failures of the public health system in the face of SARS, recommending the need to improve the PH system, including local and provincial preparedness, core competencies, and development and skill-building related to public health competencies to support public health professionals to address current and evolving health issues. Documents in both provinces also highlight the unique context of PH planning [27,43]. While governments had developed HHR policy documents, mostly in response to general shortages of nurses and physicians [20,45], many provincial HHR strategies lack a PH component despite the stated desire to strengthen the PH workforce, ensure equity, avoid resource duplication, and enhance coordination and capacity building. A key strategy identified in the documents was the importance of PH collaborating with governments and other partners beyond the immediate health system to address systemic and workforce issues [27,31]. A responsive public health system requires coordinated planning between systems and HHR. Strategic public health policy development and implementation needs to consider the systemic as well as PHHR issues to achieve policy goals [11].

### Populations of concern/ priority populations

BC and ON documents prioritized the importance of the social and environmental determinants of health in reducing health inequities [15,16,52]. Documents in both provinces emphasized the importance of considering inequity and population health perspectives in moving public health priorities forward [16,54]. Both provinces also spoke of targeting programming to meeting local need and of developing programmes to address characteristics (for example, food, early childhood development) in specific contexts and settings (for example, schools, gardens, and so on). Both the BC Core Public Health Functions and Ontario Public Health Standards discuss the importance of public health addressing the specific needs of those populations considered vulnerable or at-risk; BC refers to *populations of concern* or *vulnerable populations* while ON uses the term *priority populations*. In a separate analysis, researchers examined BC and ON public health renewal documents for how they conceptualized health equity. They identified that while both provinces defined health inequities in a similar way, how health equity was discussed in policy documents differed [55].

In BC, health authorities (which include public health) may conduct a gap analysis often based on population health status assessments; these assessments can aid in identification of populations of concern and health issues that need to be addressed through public health programmes and services [56]. In addition, the use of both a population health and equity lens are explicitly described as fundamental to the core functions and contribute to identification of populations of concern. One example of a priority population for public health programmes and services in BC is First Nations/Aboriginal peoples reflecting that province’s context [15,57] including development of a First Nations health plan of which HHR is a critical component [35]. Most BC documents explicitly identified First Nations/ Aboriginal peoples, and strongly emphasized the importance of developing culturally-appropriate programmes in partnership with Aboriginal communities and other diverse groups such as immigrants and refugees [31]. BC documents recognized that tailored programmes specifically supporting Aboriginal health and health determinants are essential, and that these programmes will need to be flexible to accommodate the diversity of contexts, communities, and governance structures that exist. In terms of HHR, BC also explicitly recognized that Aboriginal health supports are often overlooked in the public health sphere because they tend to fall outside of provincial health care jurisdiction.

The ON documents defined priority populations broadly, as those populations identified by surveillance, epidemiological or other research studies, that are at risk and for which public health interventions may be reasonably considered to have a substantial impact at the population level [16]. In this approach to identifying priority populations, a population health lens is explicitly discussed while the application of an equity lens is assumed in the use of terms such as ‘health inequities’. However, no documents specify the use of an equity lens to identify priority populations. In ON, low-income individuals and Aboriginal populations were also identified as examples of priority populations; however, the focus on Aboriginal populations is less obvious in ON documents. In ON, each public health unit is responsible for identifying priority populations specific to their context and jurisdiction [16]. For both BC and ON, the specific local level priorities would not necessarily be captured in provincial policy documents.

## Conclusions

Public health renewal in BC and ON has highlighted significant issues in the public health workforce. Policy documents from government and related public health organizations in both provinces have identified similar key issues in planning the public health workforce. These include the importance of education, training, and competencies required to meet BC Core Public Health Functions/ON Public Health Standards, ensuring a sufficient supply of needed PHHR, developing public health leadership, ensuring public health capacity exists to address significant events, addressing the unique needs of priority populations/populations of concern, and the importance of intersectoral collaboration. HHR planning in general has shifted away from a narrow focus on supply or numbers of healthcare providers to focus more on identification of population health needs and addressing health inequities, and the competencies required to meet these needs [20]. This shift is evident in many of the public health policy documents reviewed with much of the discussion focused on core public health competencies, education and training, and professional development as important elements to support the public health workforce. The delivery of effective and efficient public health programme and services requires consideration of both the competence and capabilities of the workforce [39]. These provincial policy documents also align with national strategic directions emphasizing core competencies unique to public health [10,58]. Similarities in BC and ON PHHR policy directions provide common ground for collaborative work, building on the national PHHR policy vision [10]. This collaboration may reduce duplication and more efficiently use financial and human resources.

Researchers and policy-makers have called for HHR planning approaches that are based on population health needs rather than utilization trends [10,20]. Both provinces have identified the importance of planning public health services based on identifying regional/local population health needs and have incorporated these into their provincial policies [16,59]; this may provide needed information to support planning of PHHR based on assessed population health needs within regional health authorities or local health boards. Planned analysis in this programme of research includes a review of public health workforce plans.

While many similarities exist between the provinces, the context distinctive to each province has influenced and shaped how they have focused their PHHR policies. Clearly, the SARS event had a considerable impact on PHHR policy discussions in ON, particularly in the mid-2000s with recommendations for PHHR strongly linked to SARS-related issues such as surge capacity, lack of collaboration between sectors, and shortages of some public health personnel such as Medical Officers of Health and infection control specialists. For BC, the early focus on essential public health functions influenced PHHR policy and placed greater emphasis on the competencies required to meet core functions. Furthermore, given the differences between the provinces in how public health is delivered, PHHR policy must be context specific [11]. BC and ON differ in how PH is situated in the broader health care system, whether integrated into health authorities in BC or more independent and autonomous in ON, this has implications for PHHR planning. The unique contexts in BC and ON were evident in the findings; aspects of PHHR were emphasized differently in policy development and implementation.

This policy analysis identified progressive work on PHHR policy and planning with early documents providing an inventory of PHHR issues to be addressed and later documents providing evidence of beginning policy development and implementation. In recent months, ON published their provincial strategic plan for PH [60] and BC has published its updated guiding framework for PH [59]. Both provinces’ documents highlight the importance of PHHR to achieve strategic directions in PH. However, while much policy-related work has been conducted to enhance PHHR planning in both provinces, there has been little evaluation of the effectiveness of the PHHR strategies. Looking ahead, future analyses should examine implementation of provincial PHHR policies at the local or regional level to examine the extent to which strategic directions are achieved. A key focus of the RePHS study is to examine the impact of provincial policies on the public health workforce at the public health unit or regional level in both provinces further enhancing understanding of PHHR policy and planning.

## Abbreviations

BC: British Columbia; HHR: health human resources; MPH: Master’s of public health; ON: Ontario; PH: public health; PHAC: Public Health Agency of Canada; PHHR: public health human resources; RePHS: Renewal of Public Health Systems; SARS: severe acute respiratory syndrome.

## Competing interests

The authors declare that they have no competing interests.

## Authors’ contributions

SR led all aspects of the project including development of the coding framework, data analysis and interpretation, and preparation of the manuscript. MM led the conceptualization of the overarching RePHS research project and the proposal development; participated with the larger RePHS team in conceptualizing the policy review described in this paper; assisted with development of the coding framework; and contributed to interpreting the data and editing the draft. DA conducted the data analysis and interpretation. CM and NPJ assisted with development of the coding framework, identification of documents, and interpretation of coded text. All authors reviewed and approved the final manuscript.

## Authors’ information

SR is an Assistant Professor, Western University, Arthur Labatt Family School of Nursing. She has expertise in health human resources policy and planning.

## Supplementary Material

Additional file 1 Documents Analysed. Click here for file

Additional file 2 Coding Template. Click here for file

## References

[B1] Frank J Di Ruggiero E Public health in Canada: what are the real issues C J Public Health 2003 94 3 190 192 10.1007/BF03405064 PMC6979598 12790492

[B2] Institute of Medicine The Future of the Public’s Health in the 21st Century 2002 Washington, DC: The National Academies Press

[B3] National Advisory Committee on SARS and Public Health Learning from SARS – Renewal of Public Health in Canada. A report of the National Advisory Committee on SARS and Public Health 2003 Ottawa, ON: Health Canada Report No.: 1210

[B4] European Centre for Disease Prevention and Control Annual report of the Director: 2005 2005 Stockholm: Author

[B5] World Health Organization The World Health Report 2006 - Working Together for Health 2006 Geneva, CHE: World Health Organization

[B6] Chambers LW Sullivan SM Reflections on Canada’s public health enterprise in the 21st century Healthcare Papers 2007 7 3 22 30 10.12927/hcpap.2007.18754 17476125

[B7] Tilson H Berkowitz B The public health enterprise: examining our twenty-first-century policy challenges Health Aff 2006 25 4 900 910 10.1377/hlthaff.25.4.900 16835168

[B8] Gotway Crawford CA Summerfelt WT Roy K Chen Z Meltzer DO Thacker SB Perspectives on public health workforce research J Public Health Manag Pract 2009 15 6 S5 S15 19829231 10.1097/PHH.0b013e3181bdff7d

[B9] Moore J Studying an ill-defined workforce: public health workforce research J Public Health Manag Pract 2009 15 6 S48 S53 19829230 10.1097/PHH.0b013e3181b23978

[B10] Joint Task Group on Public Health Human Resources A Pan-Canadian Framework for Public Health Human Resources Planning 2005 Ottawa, ON: Government of Canada

[B11] Beaglehole R Dal Poz MR Public health workforce: challenges and policy issues Hum Resour Health 2003 1 4 10.1186/1478-4491-1-4 12904251 PMC179882

[B12] Public Health Agency of Canada About the Agency [internet] Available from: http://www.phac-aspc.gc.ca/about_apropos/index-eng.php

[B13] Government of Ontario Health Protection and Promotion Act, 2009 2009 Toronto, ON: Government of Ontario

[B14] Government of British Columbia Public Health Act, 2008 2009 Victoria, BC: Government of British Columbia

[B15] Ministry of Health Services A Framework for Core Functions in Public Health: Resource Document 2005 Victoria, BC: Government of British Columbia

[B16] Ontario Ministry of Health and Long-Term Care Ontario Public Health Standards 2008 2009 Toronto, ON: Government of Ontario

[B17] Renewal of Public Health Services in BC and Ontario [Internet] 2009 Available from: [http://www.uvic.ca/research/groups/cphfri/projects/currentprojects/rephs/index.php]

[B18] Buse K Mays N Walt G Making Health Policy 2012 2 Berkshire, UK: Open University Press McGraw-Hill

[B19] Pal LA Beyond Policy Analysis: Public Issue Management in Turbulent Times 2010 Toronto, ON: Nelson Education

[B20] Federal/Provincial/Territorial Advisory Committee on Health Delivery and Human Resources A framework for Pan-Canadian Collaborative Health Human Resources Planning 2005 Ottawa, ON: Government of Canada

[B21] Krippendorff KA Content Analysis: An Introduction to Its Methodology 2012 3 Thousand Oaks, CA: Sage

[B22] QSR International NVivo Qualitative Data Analysis Software, version 9; released in 2010

[B23] Association of Local Public Health Agencies Creating a Sustainable Public Health System in Ontario 2004 Toronto, ON: Association of Local Public Health Agencies

[B24] Public Health Association of British Columbia Context and Connections Matrix: Core and Technical Competencies for Public Health in BC Project 2010 Vancouver, BC: Public Health Association of British Columbia

[B25] Moloughney B Frank J Di Ruggiero E Revamp Canada’s public health system — and do it quickly: think-tank CMAJ 2003 169 4 325

[B26] Ontario Ministry of Health and Long-Term Care Operation Health Protection 2004 Toronto, ON: Government of Ontario

[B27] Capacity Review Committee Revitalizing Ontario’s Public Health Capacity: The Final Report of the Capacity Review Committee 2006 Toronto, ON: Author

[B28] Hollander Analytical Services Ltd Final Report of the Midterm Evaluation, and a Framework for Future Evaluations, for the Core and Technical Competencies for Public Health in BC Project 2010 Vancouver, BC: Public Health Association of British Columbia

[B29] Ontario Public Health Association and Partners Ontario Public Health Performance Management Competency Profiles 2009 Toronto, ON: Ontario Public Health Association and Partners

[B30] Ontario Public Health Association Annual Report April 1, 2004 - March 30, 2005 2005 Toronto, ON: Ontario Public Health Association

[B31] Public Health Association of British Columbia BC Map of Public Health Services 2007 Vancouver, BC: Public Health Association of British Columbia

[B32] Zena Simces & Associates Core and Technical Competencies for Public Health in BC: Phase 1 – Needs Assessment Technical Report 2008 Vancouver, BC: The Public Health Association of BC

[B33] The Public Health Human Resources Task Group Guidelines for MPH Programs in Canada 2009 Ottawa, ON: Public Health Agency of Canada

[B34] Health Human Resources Strategy Division Medical Education [internet] 2013 Available from: http://www.health.gov.on.ca/en/pro/programs/hhrsd/physicians/medical_education.aspx

[B35] Ministry of Health Services Discussion Paper to Support the First Nation’s Health Human Resources (Internal document) 2011 Victoria, BC: Government of British Columbia

[B36] British Columbia Provincial Health Officer Pathways to Health and Healing – 2nd Report on the Health and Well-Being of Aboriginal People in British Columbia Provincial Health Officer’s Annual Report 2007 2009 Victoria, BC: Government of British Columbia

[B37] Ontario Ministry of Health and Long-Term Care Final Report of the Agency Implementation Task Force: from Vision to Action: A Plan for the Ontario Agency for Health Protection and Promotion 2006 Toronto, ON: Government of Ontario

[B38] Ontario Expert Panel on SARS and Infectious Disease Control For the Public's Health: Initial Report of the Ontario Expert Panel on SARS and Infectious Disease Control 2003 Toronto, ON: Government of Ontario

[B39] Fraser SW Greenhalgh T Coping with complexity: educating for capability BMJ 2001 323 799 803 10.1136/bmj.323.7316.799 11588088 PMC1121342

[B40] Zena Simces & Associates Core and Technical Competencies for Public Health in BC: Phase 1 – Needs Assessment Interim Report for Distribution 2008 Vancouver, BC: The Public Health Association of BC

[B41] The SARS Commission Interim Report: SARS and Public Health in Ontario 2004 Toronto, ON: The SARS Commission 10.1089/153871304323146423 15225406

[B42] The SARS Commission Second Interim Report: SARS and Public Health Legislation 2005 Toronto, ON: The SARS Commission

[B43] Ministry of Health Services Enumeration of BC Public Health System Workforce Pilot Project Plan (Draft) 2011 Victoria, BC: Government of British Columbia

[B44] Quality Worklife Quality Healthcare Collaborative Within our Grasp: A Healthy Workplace Action Strategy for Success and Sustainability in Canada’s Healthcare System 2007 Ottawa, ON: Canadian Council on Health Services Accreditation

[B45] Advisory Committee on Health Human Resources Our Health, Our Future: Creating Quality Workplaces for Canadian Nurses: Final Report of the Canadian Nursing Advisory Committee 2002 Ottawa, ON: Health Canada

[B46] Public Health Association of British Columbia Backgrounder to a Proposal for a Public Health Workforce Development Network Vancouver, BC Author; ND

[B47] BC Ministry of Healthy Living & Sport Public Health Human Resources Plan 2010 2010 Victoria, BC: Government of British Columbia

[B48] Consulting S Report on Stakeholder Consultations in Public Health Units in the Province of Ontario to the Capacity Review Committee 2006 Toronto, ON: Starfield Consulting

[B49] BC Ministry of Healthy Living and Sport Model Core Program Paper: Prevention of Chronic Diseases 2010 Victoria, BC: Government of British Columbia

[B50] BC Ministry of Healthy Living and Sport Model Core Program Paper: Healthy Child and Youth Development 2010 Victoria, BC: Government of British Columbia

[B51] BC Ministry of Healthy Living and Sport Model Core Program Paper: Healthy Infant and Child Development 2009 Victoria, BC: Government of British Columbia

[B52] Public Health Association of British Columbia Consultation Document: Core and Technical Competencies for Public Health in BC 2008 Victoria, BC: Public Health Association of British Columbia

[B53] Ministry of Health and Long-Term Care Ontario Public Health Organizational Standards 2011 Toronto, ON: Government of Ontario

[B54] Public Health Association of British Columbia Update on the PHABC’s Public Health Core Competencies Project in BC 2011 Vancouver, BC: Public Health Association of British Columbia

[B55] Pinto AD Manson H Pauly B Thanos J Parks A Cox A Equity in public health standards: a qualitative document analysis of policies from two Canadian provinces Int J Equity Health 2012 11 28 10.1186/1475-9276-11-28 22632097 PMC3479047

[B56] Ministry of Health Services Public Health Renewal in British Columbia: An Overview of Core Functions in Public Health 2005 Victoria, BC: Government of British Columbia

[B57] BC Ministry of Healthy Living and Sport Model Core Program Paper: Communicable Disease 2009 Victoria, BC: BC Ministry of Healthy Living and Sport

[B58] Public Health Agency of Canada Core Competencies in Public Health in Canada (release 1.0) 2008 Ottawa, ON: Public Health Agency of Canada

[B59] Ministry of Health Promote, Protect and Prevent: Our Health Begins Here. BC's Guiding Framework for Public Health 2013 Victoria, BC: Government of British Columbia

[B60] Ministry of Health and Long-Term Care Make No Little Plans: Ontario’s Public Health Sector Strategic Plan 2013 Toronto, ON: Government of Ontario

