# Supplementary material for: Public health human resources: a comparative analysis of policy documents in two Canadian provinces
Source: Hum Resour Health. 2014 Feb 24;12:13. doi: 10.1186/1478-4491-12-13 (PMC3936858; doi:10.1186/1478-4491-12-13)
Supplement: Additional file 1 — Documents Analysed. [file 1478-4491-12-13-S1.pdf]

Additional File 1: Documents Analysed

**British Columbia**	**Ontario**
Ministry of Health Services. (2005). *Public health renewal in British Columbia: An overview of core functions in public health.* Victoria, BC: Author.	Ontario Expert Panel on SARS and Infectious Disease Control. (2003). *For the public's health: Initial report of the Ontario expert panel on SARS and infectious disease control.* Toronto, ON: Ministry of Health and Long-Term Care.
Ministry of Health Services. (2005). *A framework for core functions in public health: Resource document.* Victoria, BC: Author.	Association of Local Public Health Agencies. (2004). *Creating a sustainable public health system in Ontario.* Toronto, ON: Author.
BC Ministry of Health. (2007). *Model core program paper: Healthy communities.* Victoria, BC: Author.	Ontario Ministry of Health and Long-Term Care. (2004). *Operation health protection.* Toronto, ON: Author
BC Ministry of Health. (2007). *Model core program paper: Healthy living.* Victoria, BC: Author.	The SARS Commission. (2004). *Interim report: SARS and public health in Ontario.* Toronto, ON: Ministry of Health and Long-Term Care.
Public Health Association of British Columbia. (2007). *BC map of public health services.* Vancouver, BC: Author.	The SARS Commission. (2005). *Second interim report: SARS and public health legislation.* (). Toronto, ON: The SARS Commission.
BC Ministry of Health. (2007). *Model core program paper: Health assessment and disease surveillance.* Victoria, BC: Author.	Starfield Consulting. (2006). *Report on stakeholder consultations in public health units in the province of Ontario to the Capacity Review Committee.* Toronto, ON: Ministry of Health and Long-Term Care.
Zena Simces & Associates. (2008). *Core and technical competencies for public health in BC: Phase 1 – needs assessment interim report for distribution.* Victoria, BC: The Public Health Association of BC.	Ontario Ministry of Health and Long-Term Care. (2006). *Final report of the agency implementation task force: From vision to action: A plan for the Ontario agency for health protection and promotion.* Toronto, ON: Author
Public Health Association of British Columbia. (2008).<br>*Consultation document: Core and technical competencies for public health in BC.* Victoria, BC: Author.	Capacity Review Committee. (2006). *Revitalizing Ontario's public health capacity: The final report of the capacity review committee.* Toronto, ON: Queen's Printer for Ontario.
Government of British Columbia. (2008). Public Health Act. http://www.bclaws.ca/EPLibraries/bclaws_new/document/ID/freeside/00_08028_01	HealthForceOntario. (2007). *Health human resources toolkit.* Toronto, ON: Author
Zena Simces & Associates. (2008). *Core and technical competencies for public health in BC: Phase 1 – needs assessment technical report.* Victoria, BC: The Public Health Association of BC.	Government of Ontario. (2009). *Regulated Health Professions Act, 1991.* Toronto, ON: Government of Ontario.
BC Ministry of Healthy Living and Sport. (2009). *Model core program paper: Reproductive health and prevention of disabilities.* Victoria, BC: Author.	Ontario Public Health Association and Partners. (2009). *Ontario public health performance management competency profiles.* Toronto, ON: Author.
BC Ministry of Healthy Living and Sport. (2009). *Model core program paper: Communicable disease.* Victoria, BC: Author.	Ontario Ministry of Health and Long-Term Care. (2009). *Infectious diseases protocol.* (). Toronto, ON: Ontario Ministry of Health and Long-Term Care.
Health Employer's Association of British Columbia. (2009). *Health human resource profile of selected public health occupational groups in British Columbia.* Vancouver, BC: Author.	Ontario Ministry of Health and Long-Term Care. (2009). *Ontario public health standards 2008.* Toronto, ON: Ontario Ministry of Health and Long-Term Care.
BC Ministry of Healthy Living and Sport. (2009). *Model core program paper: Healthy infant and*	Ontario Ministry of Health and Long-Term Care. (2009). *Nutritious food basket protocol.* Toronto, ON: Author

**British Columbia**	**Ontario**
child development. Victoria, BC: Author.	
British Columbia Provincial Health Officer. (2009). *Pathways to health and healing – 2nd report on the health and well-being of Aboriginal people in British Columbia provincial health Officer's annual report 2007.* Victoria, BC: Ministry of Healthy Living and Sport.	Ontario Ministry of Health and Long-Term Care. (2009). *Population health assessment and surveillance protocol.* Toronto, ON: Author
Government of British Columbia. (2010) Health Professions Act [RSBC 1996]	Ontario Ministry of Health and Long-Term Care. (2009). *Sexual health and sexually transmitted infections prevention and control panel.* Toronto, ON: Author
Ministry of Healthy Living and Sport. (2010). *BC ministry of healthy living and sport 2009-2010 annual service report.* Victoria, BC: Author.	Ontario Ministry of Health and Long-Term Care. (2009). *Tobacco compliance protocol.* Toronto, ON: Author
BC Ministry of Healthy Living and Sport. (2010). *Model core program paper: Prevention of chronic disease.* Victoria, BC: Author.	Government of Ontario. (2009). *Health Protection and Promotion Act, 2009.* Toronto, ON: Government of Ontario.
BC Ministry of Healthy Living and Sport. (2010). *Model core program paper: Healthy child and youth development.* Victoria, BC: Author.	Agency for Health Protection and Promotion. (2009). *Strategic plan 2010-2013.* Toronto, ON: Author
Public Health Association of British Columbia. (2010). *Context and connections matrix: Core and technical competencies for public health in BC project.* Vancouver, BC: Author.	Agency for Health Protection and Promotion. (2010). *Annual report. 2008-2009.* Toronto, ON: Agency for Health Protection and Promotion.
Hollander Analytical Services Ltd. (2010). *Final report of the midterm evaluation, and a framework for future evaluations, for the core and technical competencies for public health in BC project.* Vancouver, BC: Public Health Association of British Columbia.	
Public Health Association of British Columbia. (2011). *Update on the PHABC's public health core competencies Project in BC.* Vancouver, BC: Author.	
Ministry of Health Services. (2011). *Enumeration of BC public health system workforce pilot project plan (draft).* Victoria, BC: Author.	
Ministry of Health Services. (2011). *Discussion paper to support the first nations health human resources cluster.* Victoria, BC: Author.	
Public Health Association of British Columbia. (ND). *Backgrounder to a proposal for a public health workforce development network.* Vancouver, BC: Author.	
BC Ministry of Healthy Living & Sport. (ND). *Public health human resources plan 2010.* Victoria, BC: Author.	
Health Care Leaders Association of British Columbia. (ND). *BC health leadership capabilities framework*. Vancouver, BC: Author.	
