# Supplementary material for: Public health human resources: a comparative analysis of policy documents in two Canadian provinces
Source: Hum Resour Health. 2014 Feb 24;12:13. doi: 10.1186/1478-4491-12-13 (PMC3936858; doi:10.1186/1478-4491-12-13)
Supplement: Additional file 2 — Coding Template. [file 1478-4491-12-13-S2.pdf]

Additional File 2: Coding Template

Categories	Codes	Definition
Background/context	General HHR Planning	Broad non-specific references to HHR planning
	Public Health HR specific planning	Any references to PHHR planning
	Chronic disease prevention/sexually transmitted infection prevention related HHR planning	References to chronic disease prevention/sexually transmitted infection prevention specifically
	Health authority/Public Health Unit/Provincial/ National references	References to HHR planning specific to Health authority/PHU/Province/ National
	Priority populations	Individuals/groups at greater risk for negative health consequences (e.g. aboriginal peoples, homeless, immigrant populations).
Responsibility/ Accountability – HHR Planning	Actors/Organizations	Who/what are responsible or accountable for HHR?
	Roles	What are their roles?
	Financial resources	What financial resources are identified?
	Policy development/implementation	Who is responsible/accountable for HHR policy?
Collaborations/ partnerships	Interprofessional/interdisciplinary	Between/among HHR
	Intersectoral	Between/among different sectors
	Public Health and Primary [Health] Care	Specific to PH –PC [PHC]
Policy Assumptions	Vision/principles/goals/outcomes	The context for the HHR policy – refers to what is to be achieved, what outcomes are identified/
	Challenges	Issues/problems related to HHR and planning
	Approaches/frameworks	Planning approaches – two main approaches are usually discussed: utilization-based or needs-based (e.g., population heath needs-based)
HHR planning activities/elements	Supply/HR characteristics	References to numbers of HHR (including FCE, enumeration), shortages, demographics (e.g., age, sex) and geographical distribution.
	Deployment/Utilization/Mix	Employment patterns, where HHR are working and with whom they are working
	Leadership	All levels of PHHR leadership, successorship planning
	Education/training/competencies/ scope	Education or training programs, competencies required/developed, scope of practice including legislation
	Capacity	Demands for particular HHR, context issues contributing to capacity needs/demands
	Matching HHR to health/service needs	References to new or emerging roles or service delivery models.
HHR Management	Recruitment	Issues/Strategies/activities pertaining to recruiting/obtaining PHHR
	Retention	Issues/Strategies/Activities pertaining to retaining PHHR in the current workforce
